# A novel homozygous p.Ser69Pro *SOD1* mutation causes severe young-onset ALS with decreased enzyme activity

**DOI:** 10.1007/s00415-022-11489-x

**Published:** 2022-12-06

**Authors:** Nagia Fahmy, Kathrin Müller, Peter Munch Andersen, Stefan L. Marklund, Markus Otto, Albert C. Ludolph, Nabila Hamdi

**Affiliations:** 1 Neuromuscular Unit, Faculty of Medicine, Ain Shams University, Cairo, Egypt; 2 Department of Neurology, Ulm University, Ulm, Germany; 3 Institute of Human Genetics, Ulm University, Ulm University Medical Center, Ulm, Germany; 4 Department of Clinical Science, Neurosciences, Umeå University, Umeå, Sweden; 5 Department of Medical Bioscience, Umeå University, Umeå, Sweden; 6 German Center for Neurodegenerative Diseases (DZNE), Ulm Site, Ulm, Germany; 7 Molecular Pathology Unit, The German University in Cairo, Cairo, Egypt; 8 Neuromuscular Research Unit, Neuropsychiatry Department, Faculty of Medicine, Ain Shams University, Cairo, 11566 Egypt

**Keywords:** SOD1, Homozygous, Novel mutation, Young-onset, Enzyme activity

## Abstract

**Background:**

The dose–effect of various *SOD1* mutations on SOD1 enzymatic activity offers valuable insights into ALS pathogenesis with possible therapeutic implications. Homozygous *SOD1* mutations, yet scarce, are of special interest. We report a novel homozygous *SOD1* mutation with decreased enzymatic activity and severe early onset ALS phenotype.

**Methods:**

Whole exome sequencing and targeted screening of commonly implicated genes were conducted. Repeat-primed PCR and fragment length analysis were used for *C9orf72*. Bi-directional Sanger sequencing was used for *SOD1* and other genes. SOD1 activity was measured by direct spectrophotometry. Serum neurofilament light chain level was measured by the ELLA immunoassay system.

**Results:**

The homozygous patient for a novel *SOD1* variant p.Ser69Pro showed poor SOD1 enzymatic activity (16% of controls) and an early onset ALS phenotype predominantly affecting lower motor neurons with rapid involvement of the trunk, upper limbs and bulbar muscles. The asymptomatic heterozygous relatives had at least 68% of normal enzyme activity. Level of serum neurofilament light chain was much higher (148 pg/ml) in the patient than the relatives who had normal levels (6–10 pg/ml).

**Conclusion:**

This novel mutation adds knowledge to the ALS genotype–phenotype spectrum and supports the strong dose–effect of *SOD1* mutations associated with severely decreased enzymatic activity.

## Introduction

**Fig. 1:**
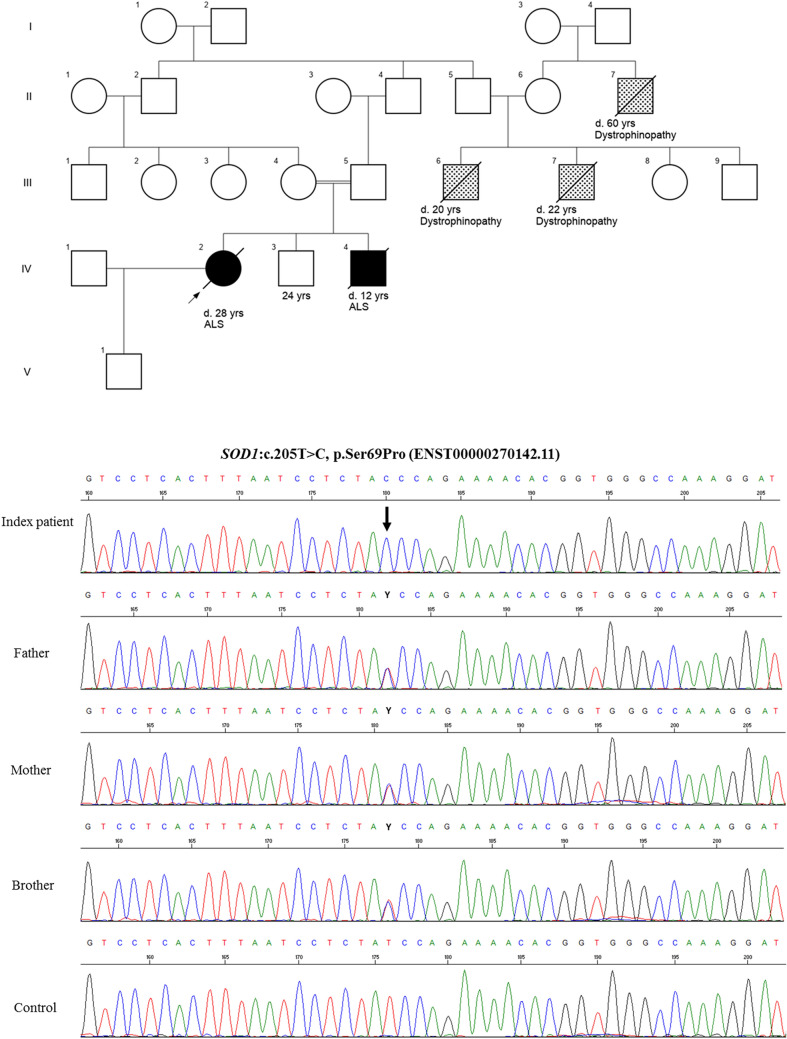
**a** Family pedigree. The proband’s parents are first-degree cousins. Her brother died at the age of 12 years most likely due to rapidly progressive juvenile ALS. Her other brother started manifesting some fasciculation in both arms. Both parents are neurologically normal. The family’s cousins and their maternal uncle possibly have dystrophinopathy (X-linked). *d*, died. **b** Genetic analysis. The proband is homozygous for the likely pathogenic variant p.Ser69Pro in the *SOD1* gene. Both parents and the proband’s brother (IV-3) are heterozygous for the same mutation

The parents of the index patient (IV-2) were cousins and had one daughter and two sons (Fig. 1). There was no history of a neuromuscular disease in the family before the youngest brother (IV-4) developed a right foot drop at the age of 10 years. Within a few months, the disease spread to the other limbs and bulbar muscles. He died from respiratory failure at the age of 12 years. No autopsy or genetic study was done. A diagnosis of juvenile amyotrophic lateral sclerosis (ALS) was postulated. His sister (IV-2) started experiencing cramps of both lower limbs upon walking or standing for longer periods at the age of 25 years. At the age of 26 years, and during her third month of pregnancy, she started complaining of weakness of her right foot rapidly progressing to foot drop. Shortly after delivering a healthy baby, a neurological examination revealed weakness of both right extensor digitorum brevis and right tibialis anterior muscles with loss of the right Achilles reflex. The left ankle reflex was preserved, while both patellar reflexes were exaggerated. A neurophysiological study was consistent with anterior horn cell disease. Within 6 months, the left lower limb was affected with foot drop and loss of the Achilles reflex. Both plantar responses were flexor, diffuse fasciculation of lower limbs, trunk and both upper limbs were observed; the tongue showed atrophy and fasciculation. Electromyography showed diffuse fasciculation with motor unit potentials of high amplitude, increased duration with frequent giant potentials and reduced interference pattern. This was found in cervical, dorsal and lumbar segments and the diagnosis of familial ALS was made based on the earlier affected brother. The condition progressed rapidly and the patient became wheelchair-bound within 3 years of onset. There was no impairment of coordination, extrapyramidal and sphincter function, or sensory deficits. Baseline ALS functional rating scale (ALSFRS**-**R) was 38, dropping to 23 after 2 years (normal range 0–48). Assessment of cognitive functions using the Edinburgh Cognitive and Behavioral ALS Screen (ECAS) after 2 years of onset gave a score of 118 (normal range 0–136), with decreased scores for language, verbal fluency, and visuospatial orientation. A brain MRI was unremarkable. The patient died 3 years after the disease onset. Her total creatin kinase (CK) level was normal. No genetic testing for dystophinopathies was done. The second brother (IV-3), in his mid-20 s, had no symptom of ALS. A recent neurological examination was unremarkable except for some infrequent fasciculations in his arms that was considered as benign. The parents, in their mid-50 s, show no neurological manifestations.

## Methods and results

Superoxide dismutase 1 *(SOD1)* mutation screening using bi-directional Sanger sequencing of all 5 exons and adjacent 30–50 bp intronic sequences revealed the patient (IV-2) to be homozygous for the likely pathogenic *SOD1* variant c.205 T > C (p.Ser69Pro). Ser69 is located close to His64 and His72 which are ligands to the enzymatically active Cu^2+^ and the stabilizing Zn^2+^ ions, respectively. Exchange to the rigid proline moiety is expected to interfere both with the metal ion charging and stability of the mutant SOD1. The parents and the unaffected brother were heterozygous for the same mutation (Fig. 1). Repeat expansion in *C9orf72* and other pathogenic or likely pathogenic variants in 43 other ALS-causing genes (including FUS, SMN was not tested) were excluded using fragment length analysis and repeat-primed PCR, and whole exome sequencing (Illumina; NexteraTM Exome Kit), respectively. The normal function of the cytosolic enzyme SOD1 is to dismute the superoxide anion radical, which is the oxygen-free radical formed in largest amount in the body. SOD1 enzymatic activity was measured with the direct spectrophotometric method using potassium superoxide [1]. The activity was related to the content of hemoglobin in blood lysates. Two sets of analysis, each comprising three superoxide additions, were carried out on blood from the probands. Analysis of an in-house control sample shows a relative standard deviation of 4.1% (*n* = 92) of the assay. The activities in human control individuals are 55.0 ± 6.5 (SD, *n* = 372) U/mg Hb. The SOD1 activity was severely decreased at 9.3 U/mg Hb in the homozygous patient. The heterozygous father, mother, and brother had moderately reduced activities at 40.3, 43.4, and 37.9 U/mg Hb, respectively. Serum NFL, measured using the ELLA system, was increased at 148 pg/ml in the patient and was normal in the father, mother, and brother at 8 pg/ml, 10 pg/ml, and 6 pg/ml, respectively (system-adopted cut-off is 45 pg/mL for ALS).

## Discussion

With the development of *SOD1*-targeting therapies for ALS patients, currently in clinical trials [2, 3], the discovery of novel *SOD1* variants is of high importance. We report on a novel homozygous *SOD1* mutation c.205T > C (p.Ser69Pro) associated with early onset of an ALS phenotype with initial asymmetrical paresis of the lower limbs, an ascending pattern of rapid disease with predominantly lower motor neuron features and later involvement of the trunk, upper limbs, and bulbar muscles. It is likely that the younger affected brother, who showed a very young age at onset and a typical ALS phenotype, was also homozygous for *SOD1* p.Ser69Pro. *SOD1* pathogenic variants in exon 3 are frequently associated with reduced disease penetrance, but the possibility that p.Ser69Pro also predisposes heterozygotes for ALS later in life cannot be excluded. As demonstrated by this case, studies in homozygous individuals may provide deeper insights into disease pathogenesis in otherwise Mendelian dominantly inherited adult-onset diseases. Only a few studies have investigated the level of SOD1 enzymatic activity in families with homozygous patients. The p.Asp91Ala and p.Leu118Val variants have normal activity and mild phenotypes with slow progression and long survival [4]. In contrast, p.Leu127Ser and p.Asn87Ser were associated with reduced enzymatic activity and young-onset. The SOD1 enzymatic activity found in the patient is the lowest activity ever reported in an ALS patient [5–7] and may have impact on therapeutic approaches with antisense nucleotides lowering SOD1 activity. Similar reports have been recently published [8]. This noticeable dose–effect of the various *SOD1* mutations on the enzymatic activity is interesting and deserves further investigation.

## Data Availability

All data relevant to the study are included in the article. For further information, contact the corresponding author (Nagia Fahmy, dr.nagiafahmy@med.asu.edu.eg, ORCID# 0000-0001-8670-0419).
